# Associations Between Selected Biological Features and Absolute and Relative Swimming Performance of Prepubescent Boys Over a 3‐Year Swimming Training Program: A Longitudinal Study

**DOI:** 10.2478/hukin-2022-0056

**Published:** 2022-09-08

**Authors:** Mariusz Kuberski, Anna Polak, Bogna Szołtys, Kamil Markowski, Ryszard Zarzeczny

**Affiliations:** 1 Chair of Physical Culture Sciences, Faculty of Health Sciences, The Jan Długosz University in Częstochowa, 42-200 Częstochowa, Poland; 2 Institute of Sport Sciences, The Jerzy Kukuczka Academy of Physical Education, 40-065 Katowice, Poland; 3 Doctoral School, The Jerzy Kukuczka Academy of Physical Education, 40-065 Katowice, Poland; 4 Institute of Health Sciences, Collegium Medicum, The Jan Kochanowski University of Kielce, 25-317 Kielce, Poland

**Keywords:** swimming performance, relative swimming velocity, physical capacity, prepubescent boys, longitudinal study

## Abstract

The study aimed to investigate the effect of 3-year swimming training on selected biological variables in prepubescent male swimmers and to determine the best predictors of absolute (VS_a_) and relative (VS_r_) swimming velocity for 50 m and 400 m front crawl. Twenty-one 10-year old boys subjected to endurance swimming training (4 x 70 min per week) and 18 boys consisting a control group were assessed semi-annually for basic anthropometric and respiratory characteristics, breath-hold time (BHT), VO_2max_, leg explosiveness (HJ), and abdominal strength endurance (ASE). After three years of training, BHT (p < 0.001), VO_2max_ (p < 0.01), HJ (p < 0.01) and ASE (p < 0.01) were greater in the swimmers than in the controls. VS_a_ and VS_r_ expressed as a percentage of baseline velocity increased more for the 50 m than for the 400 m distance (p < 0.001 and p < 0.01, respectively). The 50 m VS_a_ and VS_r_ positively correlated with those obtained for the distance of 400 m (in both cases p < 0.001). Baseline VS_a_ was negatively correlated with the increase in absolute swimming velocity for both distances (50 m: r = -0.684, p < 0.001 and 400 m: r = -0.673, p < 0.001). The best predictors of VS_a_ for 50 m and 400 m front crawl were HJ (r_2_ = 0.388; p < 0.001) and VO_2max_ (r_2_ = 0.333; p < 0.001), respectively. The key predictors of VS_r_ for both distances were age (50 m: r^2^ = 0.340, p < 0.001 and 400 m: r^2^ = 0.207, p < 0.001) and, after excluding it from analysis, HJ (50 m: r^2^ = 0.176, p < 0.001 and 400 m: r^2^ = 0.104, p < 0.001). These results suggest that regardless of prepubescent boys’ initial abilities and exercise capacity, improvement in their swimming performance mainly depends on increases in power and neuromuscular coordination.

## Introduction

Competitive swimming is a sport with the greatest number of Olympic events. In addition to consisting of four different swimming styles (front crawl, backstroke, butterfly, and breaststroke), swim distances range from 50 to 1500 m. Therefore, to be successful in competitive swimming, not only a high level of aerobic and anaerobic capacity as well as motor abilities (endurance, strength, power), but also specific somatic characteristics are required (Geladas et al., 2005; Jürimäe et al., 2007; Rejman et al., 2018). Unfortunately, sports clubs select future competitive swimmers from among children as early as aged 10 (Sousa et al., 2012), usually based on specific anthropometric criteria (low body mass, high body height, etc.) and physiological criteria (high aerobic and anaerobic capacity) (Rejman et al., 2018). These characteristics correlate significantly with the performance of adult swimmers, but in children they do not always translate into their swimming performance (Poujade et al., 2002). The reason for this can be twofold: 1) differences in the biological development of children, and 2) differences in their initial swimming skills and fitness. It is worth mentioning that in many cases the associations between biological variables and athletic performance come from cross-sectional studies (e.g., Geladas et al., 2005; Jürimäe et al., 2007; Saavedra et al., 2010), which assume that observing subjects of different ages in the same period and same-age over an extended period (3 years), would give similar results. Considering that children, especially those aged 10-12 years, tend to develop at an individual rate, the results of cross-sectional studies are prone to inaccuracies. To the best of our knowledge, there are no studies evaluating changes in the correlations between the biological characteristics of prepubescent swimmers and their performance over several years and comparing them with non-athlete controls with the same swimming background.

Regarding initial swimming skills and initial fitness of young swimmers as a likely cause of the discrepancies between their physical and somatic characteristics and swimming performance, the significance of the association between initial fitness and the effect of physical training is indicated by many authors. For instance, appropriate training can increase maximal oxygen uptake in persons with low VO_2max_ by a much greater margin than in trained or physically active subjects (Skinner et al., 2001). Analogously, swimming training may have a smaller effect on children with a high initial somatic or fitness level than those who start from a lower level. What seems to support this hypothesis is the fact that the screening process for competitive swimming tends to focus on the aforementioned characteristics of future athletes. It is, therefore, not known whether, in the absence of a screening, the improvement in swimming performance (absolute and relative swimming velocities) and the contributions from biological characteristics that frequently develop at a different rate are the same as reported by cross-sectional studies investigating relationships between prepubescent children’s biological characteristics and their absolute swimming performance (Geladas et al., 2005; Jürimäe et al., 2007; Saavedra et al., 2010).

Given the above findings and the paucity of pertinent studies, this investigation was designed: 1) to assess the effect of 3-year endurance swimming training on selected biological characteristics of prepubescent boys recruited for competitive swimming without preselection; 2) to determine which of the characteristics are the best predictors of absolute and relative velocity for 50 m and 400 m front crawl.

## Methods

### Participants

Thirty-nine boys volunteered to participate in the study. All of them had basic swimming skills, as they had two swimming lessons of 45 min per week in grades 1-3 of the primary school as part of their physical education program. Twenty-one of them, who were recruited by swimming sports clubs at the end of the third grade without any selection criteria, formed the experimental group. The other boys (n = 18), comparable with those in the experimental group in terms of age and body mass, were included in the control group. Unlike the experimental (swimming) group boys who trained in sports clubs over the study period (spanning grades 4-6), the control group did not participate in any additional physical activity. Both groups had standard physical education classes (on land) at school.

The maturity offset (MO) of the participants was calculated based on their age and body height according to the formula proposed by Moore et al. (2015). The characteristics of the study participants are presented in Table 1.

**Table 1:** Arithmetic means (±SD) or medians (IQR) of the analyzed variables in the control group (con; n = 18) and the experimental group (swim; n = 21) calculated from successive measurements and the results of two-way ANOVA with repeated measures.

Variable		Measurement						F for	F for time	F for
		1	2	3	4	5	6	group		interaction
age	con	10.472	10.940***	11.419***	11.898***	12.366***	12.887***			
[years]		±0.299	±0.282	±0,286	±0.284	±0.297	±0.283	1.1	14937.9	1.6
	swim	10.541	11.047***	11.505***	12.003***	12.489***	12.980***	n.s.	*p <* 0.001	n.s.
		±0.294	±0.287	±0.300	±0.280	±0.301	±0.304			
MO	con	-2.524	-2.183***	-1.867***	-1.458***	-1.034***	-0.535***			
[years]		±0.197	±0.194	±0.204	±0.221	±0.276	±0.322	0.4	2789.7	1.8
	swim	-2.577	-2.216***	-1.911***	-1.498***	-1.119***	-0.666***	n.s.	*p <* 0.001	n.s.
		±0.311	±0.327	±0.353	±0.364	±0.385	±0.416			
body mass	con	37.706	39.400**	41.406**	41.350***	46.706**	50.300***			
[kg]		±8.263	±8.040	±9.315	(13.700)	±11.594	±11.723	3.5	177.7	1.3
	swim	34.267	34.200**	36.414	38.486***	41.019***	44.076***	n.s.	*p <* 0.001	n.s.
		±5.747	(6.600)	±5.892	±5.959	±7.240	±7.661			
body height	con	1.448	1.472***	1.487**	1.522***	1.559***	1.604***			
[m]		±0.039	±0.037	±0.037	±0.047	±0.054	±0.065	2.3	445.1	2.1
	swim	1.424	1.449***	1.465**	1.499***	1.525***	1.564***	n.s.	*p <* 0.001	n.s.
		±0.063	±0.066	±0.067	±0.068	±0.069	±0.073			
BMI	con	17.898	17.720	18.665	17.660	19.100	19.452			
[kg∙m^-2^]		±3.337	(3.292)	±3.776	(4.405)	±4.090	±3.813	2.2	16.3	1.5
	swim	16.846	16.964	16.939	17.084	17.580*	17.938	n.s.	*p <* 0.001	n.s.
		±2.121	±2.128	±2.187	±2.041	±2.371	±2.225			
body FAT	con	17.871	19.459	20.925	21.904	21.940	22.846			
[%]		±6.191	±7.331	±8.780	±8.749	±10.276	±8.924	1.5	5.4	3.0
	swim	15.725	17.193	17.908	15.543	14.899	17.659	n.s.	*p <* 0.001	*p <* 0.05
		(7.805)	±6.535	±6.562	(7.970)	(8.803)	±6.749			
HR_rest_	con	84.000	82.222	83.556	83.111	84.000	83.333			
[beats∙min^-1^]		±8.568	±7.788	±9.294	±6.936	(8.000)	±5.531	0.7	1.9	2.5
	swim	86.857	88.000	88.000	84.000	84.000	81.905	n.s.	n.s.	*p <* 0.05
		±6.215	(4.000)	(8.000)	(0.000)	(4.000)	±5.309			
VC	con	2.082	2.157	2.197	2.197	2.212	2.218			
[l]		±0.338	±0.372	±0.328	±0.351	±0.342	±0.351	3.8	36.1	13.8
	swim	2.066	2.205*	2.359^**^	2.480^**^	2.537	2.588	n.s.	*p <* 0.001	*p <* 0.001
		±0.377	±0.325	±0.337	±0.300	±0.295	±0.296			
FEV1	con	1.677	1.647	1.678	1.673	1.677	1.656			
[l]		±0.402	±0.385	±0.423	±0.458	±0.430	±0.410	0.1	3.8	4.5
	swim	1.606	1.653	1.658	1.700	1.748	1.807	n.s.	*p <* 0.01	*p <* 0.001
		±0.387	±0.322	±0.323	±0.340	±0.316	±0.308			
BHT	con	38.720	38.936	36.913	37.050	37.338	38.867			
[s]		±11.181	±9.682	±11.033	±11.988	±10.929	±10.810	44.4	14.9	16.3
	swim	49.209	51.697	56.498	66.127*** ^a^	69.972^a^	74.199^aa^	*p <* 0.001	*p <* 0.001	*p <* 0.001
		±14.491	±12.823	±13.921	±17.302	±15.358	±14.533			
ASE	con	32.076	32.382	31.177	31.272	26.095	29.385			
[s]		±13.404	±12.171	±12.601	±13.445	(11.638)	(9.180)	9.2	2.8	7.8
	swim	40.838	41.700	39.520	43.360	49.741^*^	52.494	*p <* 0.01	*p <* 0.05	*p <* 0.001
		±18.640	±16.232	±12.803	±14.178	±16.161	±15.602			
VO_2max_	con	42.804	44.268	45.351	45.050	47.490	47.163			
[ml∙kg^-1^∙min^-1^]		±2.823	±3.506	±4.963	±5.068	(8.155)	(6.987)	9.7	13.5	6.0
	swim	46.444	46.935	48.702	48.983	51.917***	54.236	*p <* 0.01	*p <* 0.001	*p <* 0.001
		±4.143	±4.737	±6.728	±5.973	±6.257	(10.162)			
HJ	con	1.454	1.483	1.499	1.650***	1.690	1.775			
[m]		±0.191	±0.219	±0.281	±0.197	±0.203	(0.163)	8.1	71.7	7.0
	swim	1.455	1.670***	1.743**	1.798	1.867	1.911	*p <* 0.01	*p <* 0.001	*p <* 0.001
		±0.189	(0.190)	±0.130	±0.170	±0.161	±0.135			

^*^ - significantly different from the preceding measurement (^*^ -p < 0.05; ^**^ -p < 0.01; ^***^ -p < 0.001) *^a^ – significantly different from the controls’ result at the same measurement (^a^ –p < 0.05; ^aa^ –p < 0.01; ^aaa^ – p < 0.001)* *Abbreviations: MO- maturity offset; BMI - body mass index; HR_rest_ - resting heart rate; VC - vital capacity; FEV1 - forced expiratory volume in the 1 s; BHT - breath hold time; ASE - abdominal strength endurance; VO_2max_ - maximum oxygen uptake; HJ - horizontal jump*

The boys and their parents were informed about the purpose and methodology of the study and gave written consent to participate in it, as required by the Declaration of Helsinki. The protocol of the study was approved by the Bioethics Committee.

### Design and Procedures

Testing and measurements were performed every 6 months (April, October) between 8 and 12 in the morning over a period of 3 years. Anthropometric and respiratory variables were measured in the lab and the motor ability tests were carried out in the gym. Participants’ resting heart rate (HR_rest_) was measured in the lab after they rested for 15 minutes in a sitting position (to avoid potential confounders such as previous exertion, brisk walking or anxiety) by palpating the carotid artery and taking a pulse count for 15 s and multiplying the result by 4. Body mass and height that were measured with accuracy of 0.1 kg and 0.5 cm, respectively (WPT 150,0; RadWag; Poland), were used to calculate participants’ body mass indexes (BMI) by dividing body mass by the square of the body height (in meters). Heart rate was measured in a sitting position by pulse palpation after 15 min of rest (HR_rest_). Body fat content was assessed from skinfold measurements (Harpenden, M2 TOP, Käfer, Germany) using a formula proposed by Slaughter et al. (1988). Vital capacity (VC) and forced expiratory volume in the first second (FEV1) were determined with a VF-S spirometer (PELAB, Poland). The breath-hold time (BHT) was measured at the peak inspiratory flow after 10 s of hyperventilation. Spirometry was repeated three times at 5 min intervals and the best result was taken for further analysis. Maximum oxygen uptake (VO_2max_) was estimated from the results of the Maximal Multistage 20-m Shuttle Run Test (Leger et al., 1988). Abdominal muscle strength endurance (ASE) was assessed based on the results (s) of a horizontal scissors test (Chwałczyńska et al., 2017) performed by the participants in a supine position with the lower extremities elevated at an angle of 30 degrees. Explosive strength was determined from the horizontal jump test (HJ). Participants were allowed three attempts and the longest distance measured from the take-off line to the nearest point of contact at landing (back of the heels) was taken for analysis (Geladas et al., 2005). Participants performed one randomly selected test per day with at least one day rest in between.

Swimmers also performed 50 m and 400 m front crawl tests in a 25 m indoor swimming pool in the morning hours. Each test was preceded by a 10-min land warm-up, followed by a warm-up in the pool including a 300 m front crawl swim at low intensity. Having completed the warm-up, participants exited the pool and performed a given test, which started with a jump off the block. Their heart rate (HR_test_) was measured in the pool on test completion to determine exercise intensity. HR_test_ was also ascertained by palpating the carotid artery, but a pulse count was taken for 10 s and converted to beats per minute.

The training macrocycle was planned in line with the guidelines of the British Swimming Federation for boys aged 9-12 years (Lang and Light, 2010) and consisted of four training sessions of 70 min per week held in the morning. The ratio of aerobic exercises to anaerobic exercises during a session was 80 to 20%. The distances swam by participants in the three years of observation were ca. 1500 m, 2000 m, and 2500 m per session.

### Statistical Analysis

The data were tested for normality of distribution using the Shapiro-Wilk test. In the event distributions were not normal, they were transformed into logarithms for statistical analysis. The statistical significance of differences between variables characterizing the swimmers and the controls was determined using a two-way ANOVA with repeated measures for one factor (time). The swimming tests’ results represented by the mean swim velocities were evaluated by one-way ANOVA with repeated measures. When the ANOVAs pointed to the significance of the main effect, the Newman-Keuls post-hoc test was applied. The relative swim velocities recorded during successive tests are presented as percentage increases from the first measurement (=100%). Using the absolute and relative swim velocities, the slope of linear regression was calculated, where successive measurements corresponded to swim velocities. Between-variable correlations were assessed using Pearson’s correlation coefficients (r). To prevent type 1 error related to multiple comparisons from occurring, the Benjamini-Hochberg procedure and a False Discovery Rate of 0.1 were used as proposed by McDonald (2014). The contributions from particular variables to the swimmers’ performance were estimated by means of a stepwise multiple regression analysis with backward elimination. Only variables which significantly correlated with the dependent variable were included in the analysis. All computations were performed in Statistica 12.0 (Statsoft, Poland). The results are presented as arithmetic means and standard deviations (±SD) or, when their distributions were not normal, as medians (M) and interquartile ranges (IQR). In all cases excluding multiple comparisons (the Benjamini-Hochberg procedure), the level of statistical significance was set at *p* < 0.05.

## Results

All participants were prepubescent boys who did not significantly differ at baseline in any variable (Table 1). Within the three years of the study, the values of all variables excluding HR_rest_ showed significant increases (Table 1). The main effect of between-group differences was only significant for BHT, ASE, VO_2max_, and HJ, but the post-hoc analysis demonstrated that only at measurements 4, 5, and 6, the swimmers’ BHT was significantly greater (Table 1). Increases in VC (*p <* 0.001), FEV1 (*p <* 0.001), BHT (*p <* 0.001), ASE (*p <* 0.001), VO_2max_ (*p <* 0.001), and HJ (*p <* 0.001) were also greater in the experimental group. HR_rest_ was steadily declining (*p <* 0.05) only in the swimmers, while in the controls, greater increases in body fat content were observed (*p <* 0.05) (Table 1).

The one-way ANOVA showed significant differences (*p* < 0.001) between mean absolute velocities and between mean relative velocities measured at the six time points for both distances (Table 2). Absolute mean velocities were significantly greater for the 50 m distance than for the 400 m distance at each measurement (*p* < 0.001). Almost the same was observed for relative mean velocities; only at the 3^rd^ measurement the difference was not statistically significant (*p* > 0.05). Changes were reflected in the linear regression, of which slope was significantly greater for absolute and relative 50 m swim velocities than for the 400 m distance (*p* < 0.001 and *p* < 0.01, respectively).

**Table 2:** Absolute swimming velocity (m∙s^-1^) and relative swimming velocity (% of baseline velocity) recorded for swimmers (n = 21) during the tests, the heart rate measured immediately after the test (HR _test_), and the slope of linear regression.

	Distance			measurement				F	slope
		1	2	3	4	5	6		
	absolute	0.911 ^aaa^	1.012 *** ^aaa^	1.076 ^* aaa^	1.138 ^* aaa^	1.243 ^** aaa^	1.297 ^aaa^	47.7	0.088 ^aaa^
	[m∙s^-1^]	±0.246	±0.212	±0.210	±0.184	±0.168	(0.324)	*p <* 0.001	(0.069)
50 m									
	relative	100.000	110.759 ^aa^	116.936 ^**^	130.167 ^** a^	137.637 ^*** aaa^	142.052 ^aaa^	46.0	8.661 ^aa^
	[%]		(14.137)	(19.724)	±24.139	(46.715)	(46.615)	*p <* 0.001	(10.244)
	HR_test_	159.048	160.000	163.429 ^*^	165.429	165.333	164.857 ^aa^	6.1	-
	[beat∙min^-1^]	±12.209	±14.241	±16.470	±14.821	±14.783	±11.182	*p <* 0.001	
	absolute	0.653	0.699 ^**^	0.766 ^***^	0.784	0.800	0.822	33.3	0.033
	[m∙s^-1^]	±0.190	±0.175	±0.182	±0.156	±0.141	±0.142	*p <* 0.001	±0.022
400									
m	relative	100.000	105.039	113.885 ^***^	119.537	125.387	129.107	18.9	5.926
	[%]		(9.932)	(18.483)	(16.274)	(20.475)	(18.073)	*p <* 0.001	(3.977)
	HR_test_	168.000	161.524	166.000	162.381	161.524	158.476	2.0	-
	[beat∙min^-1^]	(20.000)	±10.600	(16.000)	±9.308	±9.075	±8.623	n.s.	

^*^ - significantly different from the preceding measurement (^*^ -p < 0.05; ^**^ -p < 0.01; ^***^ -p < 0.001) *^a^ – a comparison between the 50 m and 400 m tests (^a^ –p < 0.05; ^aa^ –p < 0.01; ^aaa^ –p < 0.001)*

Two-way ANOVA pointed out that HR_test_ values were not significantly different between the 50 m and 400 m tests (F = 1.0; *p* > 0.05). While HR_test_ measurements after each of the 400 m tests showed its values to be similar, the one-way ANOVA found a significant difference in HR_test_ values between measurements 2 and 3 (*p* < 0.05) (Table 2). Absolute swimming velocity measured at baseline was significantly and inversely associated with the slope of linear regression calculated from all velocity measurements during the 50 m test (r = -0.684; *p* < 0.001) and the 400 m test (r = -0.673; *p* < 0.001). Also, absolute 50 m swimming velocities were significantly and positively correlated with absolute velocities in the 400 m test (r = 0.825; *p* < 0.001). Similar associations were observed for relative swimming velocities (r = 0.663; *p* < 0.001). Pearson’s correlation coefficients between the biological variables and mean 50 m and 400 m swim velocities corrected for multiple comparisons using the Benjamini-Hochberg procedure are presented in Tables 3 and 4. The only variables which did not significantly correlate with absolute swimming velocities were body fat content (*p* > 0.05) for the 50 m test and body fat content and the BMI for the 400 m test (*p* > 0.05) (Table 3). Relative swimming velocities were significantly correlated with fewer biological variables, namely, age, HJ, BHT, ASE, body fat content, and VO_2max_ for the 50 m test, and age, HJ, and percentage body fat for the 400m test (Table 4).

**Table 3:** Pearson’s correlation coefficients between absolute 50 m and 400 m front crawl velocities and selected biological variables (n = 126) with adjustments for the false discovery rate of 0.1.

Variable	50 m swimming velocity – absolute values [m∙s^-1^]		variable	400m swimming velocity – absolute values [m∙s^-1^]		Benjamini-Hochberg critical value
	r	*p*		r	*p*	
HJ [m]	0.623	6.667 ∙ 10^-15^ significant	VO_2max_ [ml∙min^-1^∙kg^-1^]	0.578	1.449 ∙ 10-^12^ significant	0.008
VC [l]	0.609	3.770 ∙ 10^-14^ significant	VC [l]	0.502	2.076 ∙ 10^-9^ significant	0.017
body height [m]	0.559	1.017 ∙ 10^-11^ significant	HJ [m]	0.424	7.449 ∙ 10^-7^ significant	0.025
age [years]	0.541	6.447 ∙ 10^-11^ significant	body height [m]	0.414	1.466 ∙ 10^-6^ significant	0.033
body mass [kg]	0.536	9.931 ∙ 10^-11^ significant	ASE [s]	0.406	2.325 ∙ 10^-6^ significant	0.042
BHT [s]	0.484	9.727 ∙ 10^-9^ significant	BHT [s]	0.387	7.511 ∙ 10^-6^ significant	0.050
VO_2max_ [ml∙min^-1^∙kg^-1^]	0.448	1.485 ∙ 10^-7^ significant	body mass [kg]	0.352	5.376 ∙ 10^-5^ significant	0.058
HR_rest_ [beats∙min^- 1^]	-0.426	6.433 ∙ 10^-7^ significant	HR_rest_ [beats∙min^- 1^]	-0.331	1.502 ∙ 10^-4^ significant	0.067
ASE [s]	0.327	1.863 ∙ 10^-4^ significant	age [years]	0.321	2.442 ∙ 10^-4^ significant	0.075
BMI [kg∙m^2^]	0.278	1.630 ∙ 10^-3^ significant	FEV1 [l]	0.223	1.211 ∙ 10^-2^ significant	0.083
FEV1 [l]	0.254	4.086 ∙ 10^-3^ significant	BMI [kg∙m^2^]	0.141	1.154 ∙ 10^-1^ n.s.	0.092
body fat [%]	0.029	7.471 ∙ 10^-1^ n.s.	body fat [%]	-0.022	8.082 ∙ 10^-1^ n.s.	0.100

*For abbreviations: see Table 1*

**Table 4:** Pearson’s correlation coefficients between relative 50 m and 400 m front crawl velocities and selected biological variables (n = 126) with adjustments for the false discovery rate of 0.1.

Variable	50 m swimming velocity – relative values [%]		variable	400 m swimming velocity – relative values [%]		Benjamini-Hochberg critical value
age [years]	0.583	7.779 ∙ 10^-13^ significant	age [years]	0.455	8.615 ∙ 10^-8^ significant	0.008
HJ [m]	0.420	9.788 ∙ 10^-7^ significant	HJ [m]	0.322	2.379 ∙ 10^-4^ significant	0.017
BHT [s]	0.270	2.250 ∙ 10^-3^ significant	body fat [%]	-0.206	2.083 ∙ 10^-2^ significant	0.025
ASE [s]	0.223	1.227 ∙ 10^-2^ significant	BMI [kg∙m^2^]	-0.149	9.672 ∙ 10^-2^ n.s.	0.033
body fat [%]	-0.217	1.478 ∙ 10^-2^ significant	body height [m]	0.139	1.207 ∙ 10^-1^ n.s.	0.042
VO^2^max [ml∙min^-1^∙kg^-1^]	0.192	3.085 ∙ 10^-2^ significant	FEV1 [l]	0.121	1.761 ∙ 10^-1^ n.s.	0.050
HR^rest^ [beats∙min^-1^]	-0.136	1.302 ∙ 10^-1^ n.s.	VC [l]	0.119	1.852 ∙ 10^-1^ n.s.	0.058
body height [m]	0.119	1.838 ∙ 10^-1^ n.s.	BHT [s]	0.080	3.758 ∙ 10^-1^ n.s.	0.067
VC [l]	0.092	3.046 ∙ 10^-1^ n.s.	HR^rest^ [beats∙min^-1^]	-0.075	4.009 ∙ 10^-1^ n.s.	0.075
BMI [kg∙m^2^]	-0.092	3.079 ∙ 10^-1^ n.s.	VO^2^max [ml∙min^-1^∙kg^-1^]	0.063	4.802 ∙ 10^-1^ n.s.	0.083
FEV1 [l]	0.082	3.607 ∙ 10^-1^ n.s.	ASE [s]	0.048	5.910 ∙ 10^-1^ n.s.	0.092
body mass [kg]	0.009	9.242 ∙ 10^-1^ n.s.	body mass [kg]	-0.018	8.409 ∙ 10^-1^ n.s.	0.100

*For abbreviations: see Table 1*

The multiple regression analysis of absolute swimming velocities as dependent variables pointed out that the results of the HJ test and the levels of VO_2max_ were the key biological variables determining swimming velocities in the 50 m and 400 m tests, respectively (Table 5). Considering relative swimming velocities, the most important independent variable for performance in both tests was age (Table 5). Since the study lasted three years, the multiple regression analysis of 50 m and 400 m swimming velocities as dependent variables was also conducted omitting participants’ age. Then, the key independent variable determining relative swimming velocities in both tests was the result of the HJ test (Table 5).

**Table 5:** Multiple regression results for absolute (m∙s^-1^) and relative (%) 50 and 400 m swimming velocities as dependent variables.

Dependent	variable	r^2^	SEE	independent variable	ß ±SE of ß	B ±SE of B	*p*
				intercept	-	-0.224	<0.001
	absolute	0.388	±0.079			±0.030	
	value	*p* < 0.001		log HJ	0.623	1.101	<0.001
	[m∙s^-1^]				±0.070	±0.124	
				intercept	-	0.406	n.s.
	relative value	0.340	±0.072			±0.211	
log 50 m	[%]	*p* < 0.001		log age	0.583	1.577	<0.001
					±0.073	±0.197	
				intercept	-	1.939	<0.001
	relative value	0.176	±0.081			±0.031	
	without age	*p* < 0.001		log HJ	0.420	0.651	<0.001
	[%]				±0.081	±0.126	
				intercept	-	-2.381	<0.001
	absolute	0.333	±0.141			±0.398	
	value	*p* < 0.001		log VO_2max_	0.577	1.856	<0.001
	[m∙s^-1^]				±0.073	±0.236	
				intercept	-	0.962	<0.001
	relative value	0.207	±0.066			±0.194	
log 400 m	[%]	*p* < 0.001		log age	0.445	1.035	<0.001
					±0.080	±0.182	
				intercept	-	1.970	<0.001
	relative value	0.104	±0.071			±0.027	
	without age	*p* < 0.001		log HJ	0.322	0.419	<0.001
	[%]				±0.085	±0.111	

*Abbreviations: see Table 1*

## Discussion

Experimental research naturally tends to use as few variables as possible which might affect their outcome, but the approach is not always feasible. An exception includes studies with children where two factors need to be considered at the same time: the intervention and the process of biological development of the subjects. In this study, a significant difference between the swimmers’ and controls’ VO_2max_ (the main effect of group) was recorded at the end of the study (13.6% vs. 3.1%), even though none of the semi-annual measurements showed the groups to have significantly different VO_2max_.

In regard to adults, VO_2max_ induced by endurance training tends to range from 15 to 20% of its pre-training value. With respect to the effect of physical training on VO_2max_ in prepubescent children, the results of studies are inconclusive. Investigations into the influence of endurance training on VO_2max_ estimate its increase of 5-6% at the most. In studies with a significant training effect, this range rises to 8-10% (Baquet et al., 2003), which is comparable with results obtained in this study. Why physical activity increases prepubescent children’ VO_2max_ to such a small extent is not clear. The genotype accounts for around 30% of the response to physical training and physical activity and a number of other factors influence remaining 70%. It is argued, however, that although children frequently engage in physical activity, it is neither long nor intense enough to increase their VO_2max_ (Baxter-Jones and Maffulli, 2003). In this study, a significant difference in VO_2max_ of the swimmers and the controls was also confirmed by longer breath-holding times in the first group; BHT is known to be significantly associated with aerobic capacity (Cherouveim et al., 2013). Better performance of swimmers on the BHT test has a physiological explanation: swimming requires holding the breath at regular intervals, which results in momentary, recurrent hypoxia. Since physical exercise also significantly increases the production of CO_2_ and thus its blood partial pressure, it is possible that swimmers could hold their breath longer because their chemoreceptors (peripheral and/or central) controlling the respiratory function were less sensitive to CO_2_ (Trembach and Zabolotskikh, 2017).

Swimmers also showed greater strength endurance and explosive strength than controls over the course of the study. This finding is consistent with reports pointing to a significant association between muscular strength and power and swimming performance (Crowley et al., 2018; Potdevin et al., 2011). During swimming training sessions, much time is spent on improving the front crawl and back crawl leg action which increases the strength of the abdominal muscles. Because the leg and internal and external pelvic muscles are the primary component of the system propelling a swimmer forward (Roelofs et al., 2017), swim training particularly focuses on the development of these muscle groups in children.

In spite of endurance training, explosive strength of swimmers (as measured by the horizontal jump) also increased and this increase was significantly greater than in controls. This finding is interesting because the available research evidence (Coffey and Hawley, 2017) shows that in adults, a biochemical signaling conflict at the molecular level in muscle cells prevents concurrent training from increasing endurance, strength, and power at the same time. In other words, endurance training alone increases VO_2max_ while preventing muscle hypertrophy, whereas resistance training works in the opposite direction. There is, however, evidence, that in prepubescent children undergoing concurrent training, strength, power, and endurance increase simultaneously (Alves et al., 2018). This is explained through the fact that in prepubescent children, unlike adults, power and strength increases depend more on neural adaptation (involving, for instance, an increase in the number of activated motor units or changes in the coordination and recruitment thereof) rather than on muscle hypertrophy (Stricker et al., 2020). It is also worth noticing that in training sessions which involve both endurance and resistance exercises, VO_2max_ increases more when endurance exercises precede resistance exercises, whereas strength gains are greater when resistance exercises are performed first (Alves et al., 2018). Presumably, this mechanism was responsible for the concurrent increases in swimmers’ VO2max, endurance strength, and explosive strength in this study, especially considering that anaerobic exercises were also part of the training sessions.

The multiple regression analysis pointed out that the best predictors of absolute 50 m and 400 m swimming velocities were the results of the HJ test and VO_2max_, respectively. These results are similar to those obtained by other authors (Geladas et al., 2005; Jürimäe et al., 2007). The finding that VO_2max_ was the predictor of absolute 400 m swimming velocity is not surprising because of the type of swimming training (stressing endurance) and the fact that swimmers took on average 8–11 minutes to complete the 400 m swim, which suggests that much of their energy was derived from aerobic metabolism. The ability of the HJ test to predict absolute swimming velocity for the distance of 50 m is quite understandable because it is a sprint distance and the HJ measures explosive strength of the lower extremities (Geladas et al., 2005) that in front crawl may contribute up to 12% of swim propulsion (Ribeiro et al., 2015). As it appears, the high correlation between HJ test results and absolute 50 m swimming velocity was mainly due to the dive off the starting block and the swim turn (Geladas et al., 2005). Given that as much as 30% of total race time may depend on the start (Cossor et al., 1999), it is likely that greater improvements in absolute and relative swimming velocities (the slope of linear regression) in the 50 m test than in the 400 m test were related to the explosive start which is far more important for sprint swimming.

An interesting finding of this study was the high and positive correlation between the absolute swimming velocities in the 50 m and 400 m tests. A high association between the 100 m and 2000 m swim times was also reported by Meckel et al. (2012) for youth, elite swimmers. Those authors suggested that this might be related to heavy aerobic training (40–50 km per week). As it seems, also in this study, aerobic metabolism may have had a considerable effect on the youth swimmers’ exercise capacity. Chia (2006) estimated that in 10–12-year-old boys performing a 30s-anaerobic Wingate test, the aerobic processes might account for as much as 45% of total energy expenditure. Because of the longer duration of the 50 m swimming test in this study (40-50s), the contribution of aerobic energy may have been even greater. This supposition is additionally confirmed by similar heart rates recorded after each test (the main effect of the group, F = 0.1; *p* > 0.05). Their levels may appear somewhat low, but maximum heart rates in swimming tend to be ca. 8-10% lower compared with running and cycling (Roels et al., 2005). It is thought that the swimmers’ HR increases less than the dry-land athletes’ HR because of the horizontal position of the swimmer, water pressure, and the “dive reflex” (Roels et al., 2005).

An important aspect of this study is the analysis of relative swimming velocities of prepubescent boys over a period of 3 years. To our knowledge, it is the first study that examines their changes over such a long time period. The relationship between athlete’s age and performance is highlighted in many studies (Jürimäe et al., 2007; Saavedra et al., 2010), but none of them analyzed factors responsible for the improvement of relative performance of prepubescent swimmers. This is quite understandable because their main purpose was to determine factors which best correlated with absolute swimming performance and could be used by the sports clubs to identify children with the greatest potential for competitive swimming. It is, however, important to note that according to the widely-used long-term athlete development strategy (LTAD) (Lang and Light, 2010), which was used as a planning framework also for training described in this study, swimming training for children should ensure an appropriate balance between skill development and performance development, which cannot be assessed without analyzing improvements in their swimming performance.

The finding that chronological age is the most important determinant of relative swimming velocity in the 50 m and 400 m tests made in this study is, to an extent, supported by the results of other studies. Alshdokhi et al. (2020) suggested a similar increase in swimming performance for backstroke swimmers in the same age group. Saavedra et al. (2010) found, after analyzing anthropometric, general, and special fitness variables and swimming techniques, that in peri-pubescent male swimmers (mean age of 13.6 years), chronological age was the most significant predictor of swimming performance. The probability that the results of this study are influenced by a different rate of biological development of its participants (early-maturers perform better than on-time and late maturers) is very low because of the negative correlation between their swimming velocity at baseline and its increases recorded over the course of the study (the slope of linear regression).

After age was removed from the multiple regression model with relative swimming velocity as the dependent variable, the result of the HJ test result became the key predictor of swimming velocities in the 50 m and 400 m tests. The exact reason for this change is not clear. Given, however, that an increased rate of performance progress of athletes, including swimmers, during adolescence is frequently related to their somatic and physiological maturity (Beunen and Malina, 1988), it seems that the maturation of the swimmers’ (and controls’) neuromuscular system improving their performance in the HJ test best explains the improvement in swimming performance of the studied boys.

The general conclusion from this longitudinal study is that 3-year endurance swimming training improved aerobic capacity, endurance, and explosive strength of prepubescent boys. The best predictors of their absolute 50 m and 400 m front crawl velocities were the results of the horizontal jump test and the level of VO_2max_, respectively. The main determinant of the percentage increase in relative swimming velocities for both distances was age and, after it was excluded from analysis, the result of the horizontal jump test. These findings suggest that regardless of prepubescent swimmers’ natural abilities and exercise capacity, the main factor determining improvement in their performance is the increase in power and neuromuscular coordination.
